# Non parasitic hypereosinophilia revealing biventricular endomyocardial fibrosis

**DOI:** 10.11604/pamj.2018.29.58.14560

**Published:** 2018-01-22

**Authors:** Abdelkader Jalil El Hangouche, Oumaima Alaika

**Affiliations:** 1 Laboratory of Physiology, Faculty of Medicine and Pharmacy of Tangier, Abdelmalek Essaâdi University, Tangier, Morocco; 2 Laboratory of Physiology, Faculty of Medicine and Pharmacy of Rabat, University mohammed V, Rabat, Morocco; 3 Cardiology Department, Faculty of Medicine and Pharmacy of Rabat, University Mohammed V, Rabat, Morocco

**Keywords:** Hypereosinophilia syndrome, endomyocardial fibrosis, cardiovascular magnetic resonance imaging

## Image in medicine

We report a case of a 7-year-old boy, without medical history, who presented a low weight-to-height ratio associated with effort dyspnea class III and hepatalgia. The physical examination objectified signs of right sided heart failure including jugular venous dilatation, congested hepatomegaly, an ascite, without peripheral oedema. At cardiac auscultation, the heartbeats were well perceived, with a breath of tricuspid regurgitation (grade 4/6). The blood pressure was 100/60 mm Hg. The pulmonary auscultation was normal. The electrocardiogram showed a paroxysmal atrial fibrillo-flutter with ventricular rate of 95 beats/min. The chest X-ray showed a cardiomegaly with an important auricles dilatation. The blood analysis objectified iron deficiency anemia and hypereosinophilia of 600/mm^3^. The liver and renal function tests were normal. The copro-parasitological examination was normal. Given the patient poor echo window, the transthoracic echocardiography hardly objectified, in two-dimension mode, dilated atria and hyperechogenic dense biventricular apical fibrosis. The systolic function of left ventricle and right ventricle was normal. The doppler echocardiography showed tricuspid and mitral restrictive inflow patterns with moderate pulmonary hypertension. The diagnosis of endomyocardial fibrosis was made and consolidated by findings of the cardiovascular magnetic resonance imaging (CMR) that objectified a restrictive cardiomyopathy with dilated atria and biventricular apical obliteration (A). In delayed enhancement sequences, we found apical sub-endocardial late gadolinium enhancement involving both the left ventricle and the right ventricle suggesting fibrosis (B) with left ventricular apical thrombus (C). The patient has received a medical therapy including diuretics and anticoagulant and was referred to undergo endocardiectomy.

**Figure 1:**
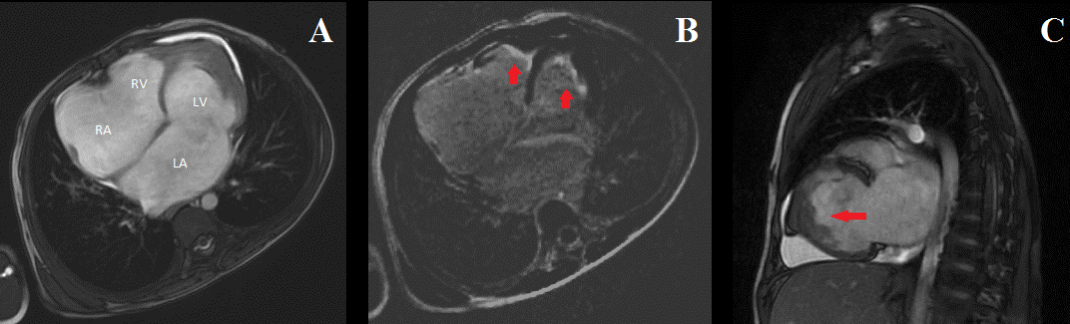
A) cine Steady State Free Precession (SSFP) four-chamber view showing dilated atria and apical bi-ventricular obliteration (LA: left atrium, RA: right atrium, LV: left ventricle, RV: right ventricle); B) inversion recovery images 10 min after contrast injection (four-chamber view) showing apical biventricular sub-endocardial late gadolinium enhancement (red arrows); C) SSFP two-chamber view showing dilated left atrium with thrombus obliterating the left ventricle apex (red arrow)

